# Specialist cancer hospital-based smoking cessation service provision in Ireland

**DOI:** 10.1007/s11845-023-03525-0

**Published:** 2023-09-23

**Authors:** Ailsa Lyons, Nancy Bhardwaj, Mouayad Masalkhi, Patricia Fox, Kate Frazer, Amanda McCann, Shiraz Syed, Vikram Niranjan, Cecily C. Kelleher, Paul Kavanagh, Patricia Fitzpatrick

**Affiliations:** 1 Department of Preventive Medicine and Health Promotion, St. Vincent’s University Hospital, Elm Park, Dublin 4, D04 T6F4, Ireland; 2 School of Public Health, Physiotherapy and Sports Science, University College Dublin, Belfield, Dublin 4, Ireland; 3 School of Medicine, University College Dublin, Belfield, Dublin 4, Ireland; 4 School of Nursing, Midwifery and Health Systems, Health Sciences Centre, University College Dublin, Belfield, Dublin 4, Ireland; 5 UCD Conway Institute of Biomolecular and Biomedical Research and UCD School of Medicine, College of Health and Agricultural Science (CHAS), University College Dublin, Belfield, Dublin 4, Ireland; 6 Health Service Executive Tobacco Free Ireland Programme, Strategy and Research, 4th Floor, Jervis House, Jervis Street, Dublin 1, D01 W596, Ireland

**Keywords:** Cessation, Health services, Prevention, Smoking topography

## Abstract

**Background:**

While much progress has been made in reducing tobacco use in many countries, both active and passive smoking remain challenges. The benefits of smoking cessation are universally recognized, and the hospital setting is an ideal setting where smokers can access smoking cessation services as hospital admission can be a cue to action. Consistent delivery of good quality smoking cessation care across health services is an important focus for reducing the harm of tobacco use, especially among continued smokers.

**Aims:**

Our objective was to document the smoking cessation medication and support services provided by specialist adult cancer hospitals across Ireland, a country with a stated tobacco endgame goal.

**Methods:**

A cross-sectional survey based on recent national clinical guidelines was used to determine smoking cessation care delivery across eight specialist adult cancer tertiary referral university hospitals and one specialist radiotherapy center. Survey responses were collected using Qualtrics, a secure online survey software tool. The data was grouped, anonymized, and analyzed in Microsoft Excel.

**Results:**

All responding hospitals demonstrated either some level of smoking cessation information or a service available to patients. However, there is substantial variation in the type and level of smoking cessation information offered, making access to smoking cessation services inconsistent and inequitable.

**Conclusion:**

The recently launched National Clinical Guideline for smoking cessation provides the template for all hospitals to ensure health services are in a position to contribute to Ireland’s tobacco endgame goal.

## Introduction

While much progress in tobacco smoking reduction has been made in many countries [1], both active and passive smoking remain a challenge. Even though Ireland has been ranked number one, jointly with the United Kingdom, in the latest (2021) Tobacco Control Scale [2] for their progress to date in tobacco control, more work needs to be undertaken to achieve the nationally stated end-game goal of a smoking prevalence of less than 5% [3]. Ireland has made substantial tobacco control progress since initially signing the World Health Organization’s (WHO) Framework Convention on Tobacco Control (FCTC) in 2003 and ratifying it in 2005 [4, 5] through the Public Health Tobacco Acts 2002–2015 [6], including banning smoking in indoor workplaces, public places, and public transportation; restrictions on tobacco advertising, marketing, promotion, and sponsorship; and standardized packaging and labeling of tobacco products. The WHO’s FCTC provides guidance to countries for implementing and managing tobacco control including the MPOWER measures (monitoring tobacco use and prevention policies; protect people from tobacco smoke; offer help to quit tobacco use; warn about the dangers of tobacco; enforce bans on tobacco, advertising, and sponsorship; and raise taxes on tobacco) [7, 8]. Totally enacting all MPOWER measures, frequent tobacco use monitoring, and increasing taxation are associated with a decrease in current tobacco smoking and thus making achieving tobacco endgame targets more likely [7]. Offering people who smoke safe, effective, and clinically sound support to stop is an MPOWER measure and one of Ireland’s priorities [3].

Smoking prevalence in the Irish general population is 18% (2021), and although an overall reduction of 5% has been seen since 2015 [3], prevalence increased from a low of 17% in 2019 to 18% in both 2021 and 2022 [3, 9, 10]. It is estimated that almost 4,500 people in Ireland die annually from smoking related diseases [3], and more needs to be done to help people who currently smoke to stop. The benefits of smoking cessation (SC) are well-established [11]. The Health Information and Quality Authority (HIQA)’s analysis of SC interventions found good evidence for use of pharmacological interventions and brief intervention, best when combined with behavioral support [12].

In Ireland, smoking-attributable hospitalizations in 2016 showed smoking-related cases consumed 309,117 bed days in publicly funded hospitals, at a cost of €172 million [13]. However, health services can also be a key setting for delivery of SC care. Irish hospital studies in 2016 and 2018 showed smoking prevalence for hospitalized patients to be 13.2% and 14.8%, respectively [14, 15]. A Cochrane systematic review found SC interventions delivered in hospital settings to be effective [16], while another found smoking bans to be effective at reducing second-hand smoke exposure [17]. The Healthy Ireland Survey findings indicate that the majority of smokers have not discussed quitting with their general practitioner during a recent consultation [9] and landmark reports on stop smoking care from both the UK Royal College of Physicians and US Surgeon General have highlighted this care gap [18, 19].

To address this gap, Ireland has recently published the National Clinical Guideline for SC which recommends that every patient/service user who engages with frontline health care professionals (HCPs) should be asked about their smoking status, the response should be documented, and every smoker should be advised to quit and be offered support at every opportunity [20]. HCPs in this context refer to all hospital staff who are working in a clinical role with direct patient contact. Hospitals are ideal settings to deliver SC interventions as patients have higher concerns for their health which may trigger a cue to action [21]. Making Every Contact Count (MECC) training is freely available to all HCPs in Ireland [22]. MECC training is behavior change training and provides HCPs with the knowledge, skills, and confidence to incorporate brief interventions into their everyday contacts with patients to address the key modifiable lifestyle behaviors related to many chronic diseases (including tobacco use). Hospital campuses are supportive of quitting because they have been smoke-free since at least 2015 [23], although compliance is not monitored. Any hospital visit or admission to a smoke-free campus [23] provides an important opportunity for HCPs to address and promote SC among patients [12].

There have been no studies to date documenting the SC medication and support services provided by specialist cancer hospitals across Ireland. The aim of our study was to document the existing SC services provided by the eight tertiary referral and specialist cancer hospitals and one specialist radiotherapy hospital in Ireland in the context of recently developed national guidelines for SC.

## Methods

A survey was developed using standards outlined in the “Stop Smoking National Clinical Guidelines No. 28 Health Services Executive (HSE)” (Section 3: Recommendations 1–3, Page 42–54) [20] and “Global Network Self-Audit Questionnaire and Planning Template,” Standard 4 [24]. The survey sought to document the availability of the following in Irish cancer hospitals: SC services and protocols, recording and availability of smoking related data, provision of SC medications, availability of SC information, provision of SC intervention training, information on e-cigarettes, and the impact of the COVID-19 pandemic on SC services.

In Ireland, all publicly provided adult cancer care is delivered through eight specialist centers, and there is one publicly funded radiation oncology network encompassing three separate sites. The survey was distributed (March–July 2021) to the eight specialist adult cancer hospitals and one specialist radiotherapy center, after obtaining approval from each hospital’s audit committee [2021]. The online survey software tool Qualtrics [25] was used, to be completed by the key relevant person in each hospital, most commonly a SC advisor or specialist, or a member of health promotion staff, with engagement from other relevant staff within the hospitals as appropriate.

SC advice referred to (i) asking patients about their smoking status, (ii) motivating them to quit, and (iii) advising them about the various SC supports available within the hospital and in the community and motivating them to use these supports; SC supports/services referred to the available in-hospital SC services. This includes any behavioral or pharmacological support, brief intervention, ongoing or intensive SC support, written materials, referral to more intensive specialist supports, and availability of intensive supports onsite. Intensive support refers to the support provided by National Center for Smoking Cessation and Training (NCSCT) practitioner-level trained smoking cessation specialists [26]. Smoking cessation practitioners assist both tobacco users and electronic cigarette users to stop smoking and/or vaping.

The information gathered was anonymized; MS Excel was used for analysis [27].

### Patient and public involvement

Patients were recruited through Patient Voice in Cancer Research (PVCR). The recruited patients were involved in the development of the survey provided to the hospitals, and the study results were shared with the patients for their opinion on completion of the study.

## Results

The survey was returned by seven specialist adult cancer hospitals and one specialist radiotherapy center (response rate 88.9%), all of whom demonstrated they had some SC related provision (88.9%). One hospital could not identify an appropriate person to complete the survey. Another hospital found it difficult to complete the survey in full due to a vacant post; as a result of which, an active in-hospital SC service was not being provided at the time.

The results are reported based on all nine hospitals approached to be included in the study. Table 1 shows a written protocol for smoking advice was available in four hospitals, a third (3) reported not having a written protocol, and two reported they were unsure or did not know. More than half (5) of the hospitals reported that all (3) or some (2) patients were asked about their smoking and provided SC advice. Most hospitals (7) reported some level of SC support or service provision, three to all patients and four to most.

**Table 1:** Summary of findings by specialist unit

**Hospital:**	**A**	**B**	**C**	**D**	**E**	**F**	**G**	**H**	**I**	**Total**
**Written protocol**	N	US	Y	N	Y	Y	Y	N	N/A	Y = 4 (44.4%) N = 3 (33.4%) Unsure or N/A = 2 (22.2%)
**All patients asked about smoking and provided SCS**	Y	US	Y	N/A	Y (not oncology patients)	Y (not outpatients)	Y	N	N/A	Y = 4 (44.4%) N = 1 (11.1%) Unsure or N/A = 3 (33.4%)
**SC support/services provided**	Most	Most	All	Most	All	Most	All	N	N/A	All = 3 (33.3%) Most = 4 (44.4%) N = 1 (11.1%) Unsure or N/A = 1 (11.1%)
**Occasions SCS provided (admission, during stay, discharge, OPD appointments, other visits (e.g., radiology), and ED)**	All	Some (other visits only)	All	Some	Some	Some	All	N/A	N/A	All = 3 (33.3%) Some = 4 (44.4%) Unsure or N/A = 2 (22.2%)
**SCS/supports provided by whom**	All	All	Some	Some	All	Some	All	N/A	N/A	All = 4 (44.4%) Some = 3 (33.3%) Unsure or N/A = 2 (22.2%)
**Types of SCS service**	All	Some	All	Some	All	Some	All	N/A	N/A	All = 4 (44.4%) Some = 3 (33.3%) Unsure or N/A = 2 (22.2%)
**Are all admitted patients offered advice, stop smoking medication, support, and referral to intensive service**	All	Some	All	Some	All	Some	All	N/A	N/A	All = 4 (44.4%) Some = 3 (33.3%) Unsure or N/A = 2 (22.2%)
**Pre-COVID-19 delivery mode of support (phone, face-to-face, group, one-to-one, and online)**	Some (no group)	Some (phone only)	Some (no group)	Some (no group)	Some (not online)	Some (no phone or group)	Some (no group or online)	N/A	N/A	Some (no group) = 3 (33.3%) Some (phone only) = 1 (11.1%) Some (not online) = 1 (11.1%) Some (no group or online) = 1 (11.1%) N = 0 (0)% Unsure or N/A = 2 (22.2%)

*Y *yes, *N *no, *US *unsure, *N/A *not applicable or no response, *SCS *smoking cessation services, *OPD* outpatient department, *ED* emergency department

In seven hospitals, the service was available in all (3) or some (4) areas: inpatient (admission, during stay, and at discharge), outpatients (first and all appointments), and other visits (e.g., attending radiology department, emergency department, and/or some other time). Some form of SC service or support was provided by all of the following list of personnel (medical, nursing, hospital SC staff, community SC staff (for patients referred on discharge), allied healthcare professionals, and others) in four hospitals, while in three, it was provided by some of these personnel. Of the following list of services (brief intervention, intensive intervention, and ongoing support), four hospitals provided all, while three provided some. When asked if all admitted patients are offered advice, stop SC, support, and referral to intensive service, four hospitals reported all were offered and three reported some were offered. Pre-COVID-19 modes of SC service delivery (phone, face-to-face, group, and one-to-one) were not all offered; however, seven delivered their service in some of these ways and/or online.

Table 2 shows the results in relation to data recording, protocols, and SC service provision. Concerning recording data on patient smoking status, household/family members smoking status, SC service referrals, interventions, who delivered the intervention, and SC medications, five hospitals recorded data on some, one recorded on all, and one did not record on any. Five hospitals record specialist SC services provided to both inpatients and outpatients, while one records data on inpatients only. The uptake by patients (inpatient, outpatient, or day-case patients) of SC services in the last year was only provided by one hospital, where it was reported as 65%.

**Table 2:** Smoking cessation service provision and data collection (9 hospitals)

**Hospital:**	**A**	**B**	**C**	**D**	**E**	**F**	**G**	**H**	**I**	**Total**
**Smoking data (patient smoking status, household/family member smoking status, SCS referrals, interventions, who delivered intervention, and stop smoking medications)**	Some	N	Some	Some	All	Some	Some	N/A	N/A	All = 4 (44.4%) Some = 3 (33.3%) Unsure or N/A = 2 (22.2%)
**Data on uptake of specialist SCS (inpatients and outpatients)**	All	Some (inpatient)	All	All	All	N	All	N/A	N/A	All = 4 (44.4%) Some = 1 (11.1%) N = 1 (11.1%) Unsure or N/A = 2 (22.2%)
**Proportion (%) of SCS uptake during the last year**	N/A	N/A	65%	N/A	N/A	N/A	US	N/A	N/A	65% = 1 (11.1%) Unsure or N/A = 8 (88.9%)
**Written protocol for stop smoking medications**	N	US	Y	Y	Y	Y	Y	N/A	N/A	Y = 5 (55.6%) N = 1 (11.1%) Unsure or N/A = 3 (33.3%)
**Availability of stop smoking medications (NRT, bupropion, and varenicline)**	All	US	All	Some (not bupropion)	Some (not bupropion)	Some (not bupropion)	Some (not bupropion)	Some (NRT only)	N/A	All = 2 (22.2%) Some = 5 (55.6%) Unsure or N/A = 2 (22.2%)
**HCP awareness of availability of protocol or stop smoking medications**	Y	N/A	US	Y	Y	US	Y	N/A	N/A	Y = 4 (44.4%) Unsure or N/A = 5 (55.6%)
**Stop smoking medications available 24 h**	Y	N/A	Y	Y	Y	US	Y	US	N/A	Y = 5 (55.6%) Unsure or N/A = 4 (44.4%)
**Stop smoking medications routinely provided (inpatients, outpatients, and day-case patients)**	Some	N/A	US	Some	Some	Some	Some	N/A	N/A	Some = 5 (55.6%) Unsure or N/A = 4 (44.4%)
**Written info on hospital website**	N (but on intranet)	Y	Y	Y	Y	Y	Y	Y	N/A	Y = 7 (77.8%) N (but on intranet) = 1 (11.1%) Unsure or N/A = 1 (11.1%)
**Info on hospital SCS, HSE Quitline, quit booklets, contact details of SCS, and hospital’s own written SC information available on website**	N	Some (hosp. SCS)	All	Some	Some (hosp. SCS’s, dept.)	Some (quit book and HSE Quitline)	Some (hosp. SCS, HSE Quitline, contact of SCS’, and own written SC info)	Some (HSE Quitline)	N/A	All = 1 (11.1%) Some = 6 (66.7%) N = 1 (11.1%) Unsure or N/A = 1 (11.1%)
**Are all patients advised of website and provided link in advance of scheduled admission/visit**	N	N	N	N	N	Y (texts and letters sent)	N	N	N/A	Y = 1 (11.1%) N = 7 (77.8%) Unsure or N/A = 1 (11.1%)
**Other methods of SCS promotion in the hospital**	Y	N	Y	Y	Y	US	Y	Y	N/A	Y = 6 (66.7%) N = 1 (11.1%) Unsure or N/A = 2 (22.2%)
**Information availability for low-literacy/speakers of other languages**	Y	N	Y	N/A	Y	US	Y	N	N/A	Y = 4 (44.4%) N = 2 (22.2%) Unsure or N/A = 3 (33.3%)
**Provision of e-cig/vaping information**	N	N	Y	Y	N	N	N	N	N/A	Y = 2 (22.2%) N = 6 (66.7%) Unsure or N/A = 1 (11.1%)

*Y *yes, *N *no, *US *unsure, *N/A *not applicable or no response, *SCS *smoking cessation services, *HCPs *healthcare professionals, *NRT *nicotine replacement therapy

A written protocol for SC medications was available in five hospitals. Only two hospitals provide all three SC medications (nicotine replacement therapy (NRT), bupropion, and varenicline); these were also the only two hospitals to provide bupropion. NRT and varenicline were provided in four hospitals; NRT only was provided in one. Four hospitals reported that HCPs were aware of the availability of a protocol on SC medications. SC medications were available 24 h a day in five hospitals.

One hospital had no written information on SC available on their website, and one did not report; of the remaining hospitals (7), one directed patients to the website for this information (via text and in letters). Other methods (e.g. business cards, posters, leaflets, and information stands on key dates such as World No Tobacco Day) were used for the promotion of SC services in six hospitals. Four hospitals reported having information available for low literacy (approved by the National Adult Literacy Agency) or speakers of other languages. Two provided information on e-cigarettes or vaping.

Making Every Contact Count (MECC) brief intervention training data were collected in four hospitals (Table 3). SC training for HCPs was reportedly available in six of the hospitals. Five hospitals had staff who are National Center for Smoking Cessation and Training (NCSCT)-trained in their hospital. Six hospitals used carbon monoxide monitoring.

**Table 3:** Staff training and COVID-19 impact on services

**Hospital:**	**A**	**B**	**C**	**D**	**E**	**F**	**G**	**H**	**I**	**Total**
**MECC training data collected**	Y	N	Y	N	Y	US	N	Y	N/A	Y = 4 (44.4%) N = 3 (33.3%) Unsure or N/A = 2 (22.2%)
**Promotion of SC training for HCPs**	Y	US	Y	N	Y	Y	Y	Y	N/A	Y = 6 (66.7%) N = 1 (11.1%) Unsure or N/A = 2 (22.2%)
**NCSCT trained staff**	Y	US	Y	Y	Y	N	Y	N	N/A	Y = 5 (55.6%) N = 2 (22.2%) Unsure or N/A = 2 (22.2%)
**Carbon monoxide monitoring**	Y	US	Y	Y	Y	Y	Y	N	N/A	Y = 6 (66.7%) N = 1 (11.1%) Unsure or N/A = 2 (22.2%)
**COVID-19 pandemic impact**	Y	Y	Y	Y	Y	Y	Y	N/A	N/A	Y = 7 (77.8%) Unsure or N/A = 2 (22.2%)

*Y *yes, *N *no, *US *unsure, *N/A *not applicable or no response, *SC *smoking cessation, *HCPs *healthcare professionals, *MECC *Making Every Contact Count, *NCSCT *National Center for Smoking Cessation and Training

The COVID-19 pandemic impacted the delivery of SC in seven hospitals; two did not report on impact. The level of impact, however, varied with some hospitals reporting staff redeployment and services put on hold. Other hospitals reported reduced services and/or a move to a virtual or telephone service. One center maintained face-to-face contact for inpatients only throughout the pandemic, with post-discharge follow-up offered exclusively by telephone.

## Discussion

The results of this survey show SC information and services are provided in some form in seven hospitals. Given that this survey was conducted in very difficult pandemic conditions when hospitals were under prolonged increased pressures, there was a good, detailed response in general. However, there is substantial variation noted, resulting in inconsistent and inequitable access to SC services for patients. With the recent (January 2022) publication and launch of the National Clinical Guideline No. 28 Stop Smoking [4], there is now a clear evidence-based standard with comprehensive recommendations for the provision of SC information and services in hospitals.

This new guidance has three clear recommendations that directly relate to the general adult population. First, all HCPs should ask about an individual’s smoking behavior. Second, all HCPs should advise all smokers about the harms of smoking for themselves and others and the benefit of quitting, and they should advise that help can be provided or arranged to support a quit attempt. Where a patient is interested in quitting, treatment needs and preferences should be discussed. HCPs should advise that making an unsupported quit attempt is less effective than using recommended supports, and treatment should be provided or arranged. Third, for people who are currently interested in quitting, all HCPs should recommend that behavioral support, either alone or in combination with pharmacological supports, increases the chances of successful quitting. Smoking status and all discussions, interventions, and outcomes should be documented.

The current research shows that only a few hospitals reported asking all patients about smoking and offering SC services, while others asked and offered to only some patients. Some of the SC offered included advice, medication, support, and access to an intensive service. Just one hospital reported recording all relevant smoking data, and just over half of all hospitals (5) had data on the uptake of specialist SC services for all.

The new guidance also recommends that varenicline, alone or in combination with NRT, is offered as a first line treatment, and if for some reason varenicline is contraindicated, combination NRT should be offered. NRT monotherapy, bupropion (alone or in combination with NRT), or nortriptyline can also be recommended as a second-line therapy. Our research did not seek information on nortriptyline as it was not at that time a recommended therapy. Just two hospitals surveyed offered the provision of all other recommended SC medications, while half of hospitals advised that bupropion was not available and none reported that SC medications were routinely provided to all patients. It is clear that the surveyed hospitals are not as yet fully compliant with the newly published National Clinical Guideline for SC.

Other recommendations within the guidance are the implementation of Tobacco Free Campus Policy [15] and the Making Every Contact Count (MECC) program [19]; capacity building for implementing recommendations is stressed. Since 2015, a Tobacco Free Campus Policy has been national hospital policy and so this was not a specific question in the audit. MECC data was collected by four hospitals; SC training was promoted in six, and in five, there were NCSCT [20] trained staff; NCSCT training provides skills to HCPs to deliver intensive SC interventions to smokers.

There are few published cost-effective studies of hospital smoking cessation services, and where they exist, they do not replicate exactly the model of specialist hospital found in Ireland; however, all studies point to the cost effectiveness of these services [28–30]. In terms of impact on smoking cessation and cancer outcomes, Frazer et al. [31] conducted a systematic review of smoking cessation interventions for patients with cancer and found a range of interventions, some with success in increasing quit rates. The link to a positive impact on health outcomes has been shown in several studies with reduced post-operative mortality, improved quality of life, and increased survival [32–34].

The results of this survey are broadly in line with similar audits and surveys conducted in the UK. This study is the first of its kind in Ireland, whereas the UK has previously investigated and reported on the provision of hospital-based SC services. Proctor et al. [35], similar to the current study, found that there was considerable variation in how SC services were staffed and run. All of the inpatient wards were able to provide NRT, and just over half offered varenicline and a third bupropion. These findings were supported by a British Thoracic Society (BTS) audit of UK hospital SC services in 2019 [36] against National Institute for Health and Care Excellence and BTS standards noting adherence to these national standards for SC was low. Neither of these UK investigations reported e-cigarette information provision, while our current study showed there was a lack of information provision across hospitals on the harms of e-cig/vaping use.

In Canada, implementation of performance monitoring of a pre-defined standard by a healthcare funder increased delivery of an evidence-based SC intervention across multiple hospitals [37]. The proportion and number of patients doubled in the 3-year period following introduction of the new policy. This is not something we see in Ireland but perhaps it deserves consideration if we are to fully address SC in our hospitals.

We have shown considerable variation in the SC information and services provided across the adult cancer specialist centers and a radiotherapy center, prior to and during the COVID-19 pandemic. We acknowledge data is collected at a national level on the number of dedicated staff engaged in SC services in each hospital and their activity data.

It is critical now that the HSE supports and progresses the implementation of these new National Clinical Guidelines, so as to address the gaps and inconsistencies in care delineated in this study. The findings presented here provide a useful baseline, which should be extended nationally and monitored over time to assure progress.

We believe it is possible to have a good practice framework on SC service in specialist hospitals. All the 8 hospitals have a similar range of patients attending, with some exceptions (transplant services and neurosurgery). The national guideline for smoking cessation forms a major part of a good practice framework. The national implementation of stop before the op policies and automatic referral of all smokers to smoking cessation services would form the basis for the remainder of a good practice framework.

In conclusion, this study demonstrates there is a need for improved and equally available smoking cessation support across all specialist hospitals; the recently launched National Clinical Guideline [20] for SC provides the template.

## Declarations

### PPI statement

There were 3 patient representatives on the research program steering committee, recruited through the Patient Voice in Cancer Research program https://www.ucd.ie/patientvoicecancer/.
