# Correction to: Specialist cancer hospital‑based smoking cessation service provision in Ireland

**DOI:** 10.1007/s11845-023-03557-6

**Published:** 2023-11-07

**Authors:** Ailsa Lyons, Nancy Bhardwaj, Mouayad Masalkhi, Patricia Fox, Kate Frazer, Amanda McCann, Shiraz Syed, Vikram Niranjan, Cecily C. Kelleher, Paul Kavanagh, Patricia Fitzpatrick

**Affiliations:** 1 Department of Preventive Medicine and Health Promotion, St. Vincent’s University Hospital, Elm Park, Dublin 4, D04 T6F4 Ireland; 2 School of Public Health, Physiotherapy and Sports Science, University College Dublin, Belfield, Dublin 4, Ireland; 3 School of Medicine, University College Dublin, Belfield, Dublin 4, Ireland; 4 School of Nursing, Midwifery and Health Systems, Health Sciences Centre, University College Dublin, Belfield, Dublin 4, Ireland; 5 UCD Conway Institute of Biomolecular and Biomedical, Research and UCD School of Medicine, College of Health, and Agricultural Science (CHAS), University College, Dublin, Belfield Dublin 4, Ireland; 6 Health Service Executive Tobacco Free Ireland Programme, Strategy and Research, 4th Floor, Jervis House, Jervis Street, Dublin 1, D01 W596 Ireland

**Correction to** **:** **Irish Journal of Medical Science (2023)** **https://doi.org/10.1007/s11845-023-03525-0**

The article “Specialist cancer hospital‑based smoking cessation service provision in Ireland”, written by Ailsa Lyons, Nancy Bhardwaj, Mouayad Masalkhi, Patricia Fox, Kate Frazer, Amanda McCann, Shiraz Syed, Vikram Niranjan, Cecily C. Kelleher, Paul Kavanagh and Patricia Fitzpatrick, was originally published electronically on the publisher’s internet portal on 23 September 2023 without open access. With the author(s)’ decision to opt for Open Choice the copyright of the article changed on 24 October 2023 to © The Author(s) 2023 and the article is forthwith distributed under a Creative Commons Attribution 4.0 International License, which permits use, sharing, adaptation, distribution and reproduction in any medium or format, as long as you give appropriate credit to the original author(s) and the source, provide a link to the Creative Commons licence, and indicate if changes were made. The images or other third party material in this article are included in the article’s Creative Commons licence, unless indicated otherwise in a credit line to the material. If material is not included in the article’s Creative Commons licence and your intended use is not permitted by statutory regulation or exceeds the permitted use, you will need to obtain permission directly from the copyright holder. To view a copy of this licence, visit http://creativecommons.org/licenses/by/4.0.

The original article has been corrected.

